# A randomized split-mouth clinical trial comparing pain experienced during palatal injections with two different computer-controlled local anesthetic delivery systems

**DOI:** 10.4317/jced.57506

**Published:** 2020-12-01

**Authors:** Anna Riba-Roca, Rui Figueiredo, Stanley F. Malamed, Josep Arnabat-Dominguez

**Affiliations:** 1 DDS. Faculty of Medicine and Health Sciences, University of Barcelona. Barcelona, Spain; 2 DDS, MS, PhD. Faculty of Medicine and Health Sciences, University of Barcelona. Barcelona, Spain. Researcher at the Idibell institute. Barcelona, Spain; 3 DDS. Herman Ostrow School of Dentistry of USC. Los Angeles, California, USA; 4 MD, DDS, MS, PhD. Faculty of Medicine and Health Sciences, University of Barcelona. Barcelona, Spain. Researcher at the Idibell institute. Barcelona, Spain

## Abstract

**Background:**

Several methods have been proposed to reduce pain during injection. The main aim to this study was to compare the pain perception in patients receiving palatal injections of local anesthesia using two different computer-controlled local anesthetic delivery systems (C-CLAD) – Dentapen® and The STA Wand®).

**Material and Methods:**

A randomized, split-mouth and simple blind clinical trial was carried out at the Dental Hospital of the University of Barcelona (Spain) involving a sample of 20 healthy volunteers. Each participant received two palatal injections in the same session (0.3 ml of 3% mepivacaine without vasoconstrictor), using The STA Wand® on one side and the Dentapen® on the contralateral side. The order of the devices and the side of the injections were randomly selected. Pain perception was recorded after each injection using a 10-cm numeric rating scale (NRS). A descriptive and bivariate analysis of the data was performed.

**Results:**

Pain perception was similar with both devices (*p*>0.05). The STA Wand® and Dentapen® groups yielded a mean pain score of 2.40 cm (standard deviation (SD) = 1.47, range 0-6) and 2.35 cm (SD 1.3, range 1-6), respectively. Most participants referred mild pain (80%), and none experienced severe pain. There were no adverse events.

**Conclusions:**

In the majority of cases (80%), both C-CLAD devices allow the administration of local anesthetics in the palatal area with mild pain. Both The STA Wand® and Dentapen® are equally effective in reducing pain perception levels for palatal injections.

** Key words:**Computer-controlled local anesthetic delivery, dental anesthesia; palatal anesthesia, The STA Wand, Dentapen.

## Introduction

Since most dental treatments might cause pain, local anesthesia is an essential tool to reduce or eliminate pain perception in the dental office. However, patients often express more fear of the injection of local anesthetics than of the dental treatment itself (1,2).

According to the American Dental Association, dental fear is the most common reason for not visiting the dentist, especially in children and teenagers, as it is associated with pain, discomfort and anxiety (2,3). In addition, anxiety and severe fear seem to raise pain perception during anesthesia and discomfort during the dental treatment, increasing the operating time and difficulty (4,5).

Local anesthesia can cause pain for various reasons, including soft tissue damage during penetration of the needle, pressure on injecting the anesthetic solution and its temperature and low pH (1). Several methods have been proposed to reduce pain during injection, such as the application of topical anesthesia, the use of small diameter needles, or the application of laser in the injection area (3,6,7). However, reducing the injection speed seems to be the most effective method for diminishing pain (2).

Although traditional syringes are still the most commonly used method to administer local anesthetics (8,9), since the mid-1990s several computer-controlled local anesthetic delivery systems (C-CLAD) have been developed to control the flow rate of the solution through the needle (6). Most C-CLAD devices are able to reduce the injection flow and maintain a constant speed considering the anatomical characteristics of the tissues (7). Most published studies show that these systems seem to afford more adequate pain control, especially when palatal injections are needed, in comparison with the traditional technique (1,3,4,6,7). However, to the best of our knowledge, no studies have compared the efficacy of the different commercially available devices. Thus, the aim of the present study was to compare pain perception in patients receiving palatal injections of local anesthesia using two different C-CLAD systems (The STA Wand® and Dentapen®).

## Material and Methods

A single-blind, split-mouth, randomized clinical trial involving 20 dental students was performed. The study was conducted at the Dental Hospital of the University of Barcelona (Spain) between April and May 2019 after obtaining approval from the local Institutional Review Board (Comitè d’Ètica i Investigació amb Medicaments de l’Hospital Odontològic Universitat de Barcelona; Protocol 33/2018). The study was designed complying with the CONSORT recommendations for clinical trials, and followed the Declaration of Helsinki guidelines. Before enrollment, all subjects were explained the objectives, implications and possible complications of the study and agreed to participate by signing an informed consent.

Sample size calculation was made with G.Power v3.1.3. (Heinrich-Heine Universität, Düsseldorf, Germany), and the clinical trial of Romero-Galvez *et al.* (10) was taken as a reference. The following parameters were employed for the power analysis: alpha = 0.05; beta = 0.2; expected pain in group of 3/10 (standard deviation (SD): 1.5); clinically significant difference of at least 1/10. The total sample size was established as 20 patients.

Healthy subjects over 18 years of age (American Society of Anesthesiology (ASA) score I and II) were included. The exclusion criteria were pregnancy, allergy or intolerance to mepivacaine or amide-type anesthetics, patients under treatment with analgesics or drugs that might affect pain perception, and any alteration at the injection site (palate).

We used 3% mepivacaine without vasoconstrictor (0.3 ml, Scandinibsa; Inibsa Dental, Lliçà de Vall, Spain, Inibsa Dental S.L.U.) and short needles 30G 0.3 × 25 mm (Monoprotect Plus; Inibsa Dental, Lliçà de Vall, Spain, Inibsa Dental S.L.U.) in all cases. Local anesthesia was administered by a single researcher (A.R.R.) with The STA Wand® (Milestone Scientific, Livingston, NJ) and Dentapen® (Juvaplus SA, Swiss Tecnology +, Switzerland) devices. The order of the devices (The STA Wand® or Dentapen®) and the injection side were randomized based on the website http:/www.randomization.com. The decision of enrolling patients in the trial was made before randomization (allocation concealment).

Before the injection, the patients were notified about the duration of the study and were asked to complete the modified dental anxiety scale (MDAS). The MDAS score ranges from 5 to 25, and patients scoring over 19 are considered to be highly anxious. The participants then received two injections, one with each device (The STA Wand® or Dentapen®), in a symmetrical location of the palate. To make sure that the participants were blinded, a black mask was placed over their eyes, and protective hearing devices were employed during the procedure, since the injection systems produced different acoustic signals.

**Figure 1:**
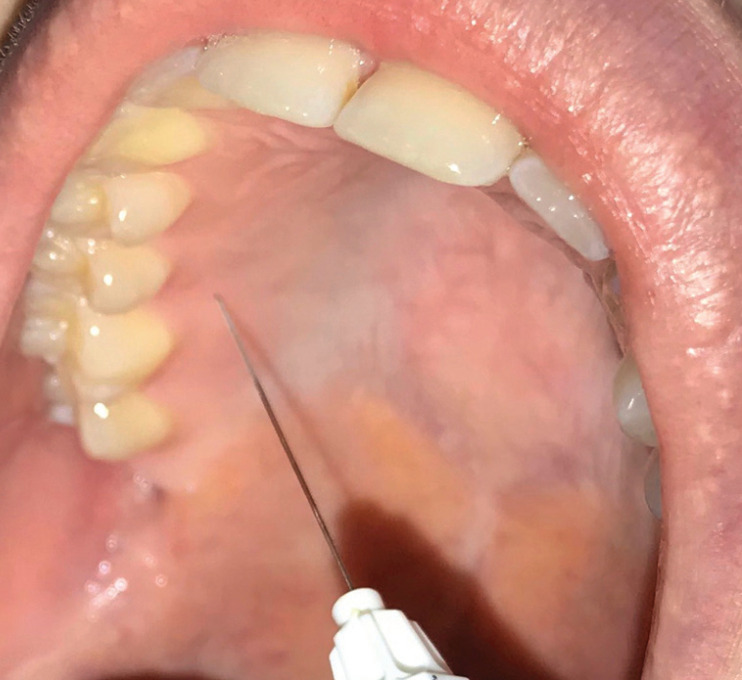
Injections were performed in the palatal zone, between the first molar and second premolar, at a distance of approximately 3 mm below the papilla.

All patients were placed in a similar position (supine position with the head tilted backwards), and topical anesthesia was not employed. Injections were performed in the palatal area between the first molar and second premolar, approximately 3 mm below the papilla (Fig. 1). The needle was always inserted with a 45-degree inclination, with the bevel facing towards the palatal tissue.

Both devices allow control of the flow rate and pressure of the anesthetic solution during injection. The STA Wand® was set with the ControlFlo speed and the Dentapen® system was used with the slow speed setting (90 seconds / ml). The programs of both devices have a comparable speed.

Immediately after each injection, the patients were asked to rate pain intensity on a horizontal 10-cm numeric rating scale (NRS) ranging from 0 (“painless”) to 10 (“worst imaginable pain”). Numeric rating scale values ranging from 0 to 3.9 were considered to be comfortable; 4 to 7 cm were considered indicative of moderate pain; and values > 7 cm were classified as severe pain.7 The pain score was regarded as the primary outcome variable.

The data were processed using the SPSS version 25 statistical package (IBM Corp.; Armonk, NY, USA). Descriptive (mean, standard deviation (SD) and ranges) and bivariate analyses were performed. After checking that the scale variables had a normal distribution (Kolmogorov-Smirnov test), paired Student t-tests were employed to compare the groups.

## Results

**Figure 2:**
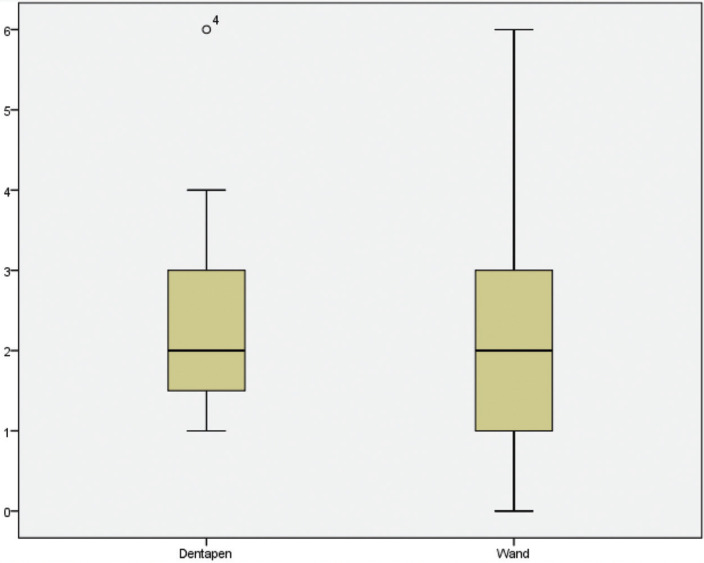
Box-plots with pain intensity scores (vertical axis) comparing both computer-controlled local anesthetic delivery systems.

A total of 20 volunteers participated in the study, and all of them were females. The mean age was 23.2 years (SD=2.5). The mean pain intensity score was of 2.40 cm (SD = 1.5; range 0-6) for The STA Wand® and 2.35 cm (SD = 1.2; range 1-6) for Dentapen® (Table 1, Fig. 2). The difference between the two devices was not statistically significant (t = -0.125; *p* = 0.902). Fifty percent of the participants referred more pain with The Wand® and 45% with Dentapen®, while 5% experienced the same pain with both devices (Table 1). None of the volunteers reported severe pain (scores > 7 cm).

**Table 1:**
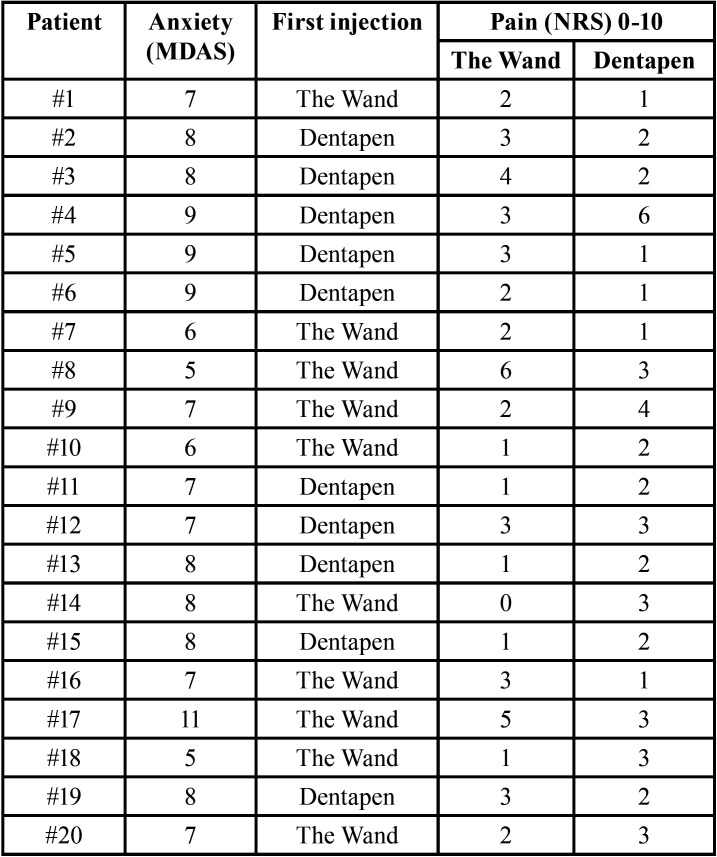
Preoperative patient anxiety assessed by the MDAS questionnaire, the initial treatment received by each participant, and the pain intensity experienced in each injection (based on a visual rating scale (VRS)). MDAS: modified dental anxiety scale; NRS: numeric rating scale.

The mean MDAS questionnaire score was 7.5 (SD = 1.43), and 20% and 80% of the participants were classified as moderately and mildly anxious, respectively (Table 1).

No adverse events were recorded in any of the groups.

## Discussion

The results of the present study show that both C-CLAD injection systems (The STA Wand® and Dentapen®) seem to yield similar outcomes in terms of pain perception during palatal injections.

C-CLAD systems are able to reduce the injection flow at a fixed pressure, regardless of variations in tissue resistance (7). This effect results in controlled, highly effective and comforTable injection even in resilient tissues such as the palate or the periodontal ligament. Maintaining an ideal flow rate of the anesthetic solution is probably the most relevant factor for ensuring comfortable injection (2). Our results seem to support this statement, since most of the patients reported mild pain. It is also important to underscore that all injections in the present study were made using a similar speed, and with the same needle caliber and local anesthetic solution.

Since both systems performed similarly in the present trial, selection of the most suitable device should be made according to the personal preferences of the clinician.

It is interesting to discuss the main differences and limitations of the two tested systems.

**Figure 3:**
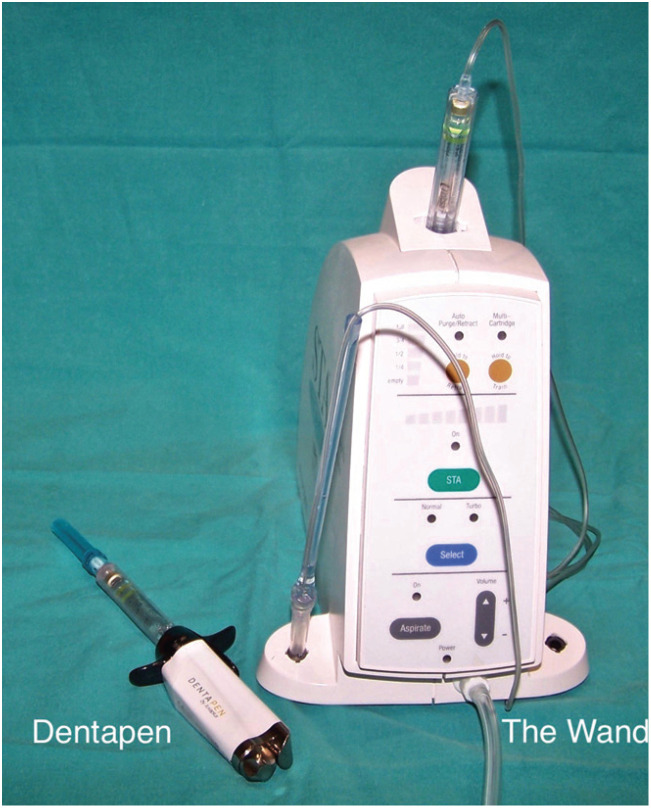
View of the Dentapen® and The STA Wand®.

The STA Wand® consists of three elements: a disposable handpiece, a computer control unit, and a foot pedal (Fig. 3). It is known for its light-weight and ergonomic handpiece, which is designed to give more tactile feedback, precision and ease to the operators. It allows focusing more on the needle position and on patient interaction, while patients find the design of the handpiece to be less threatening than the traditional syringe (8). The handpiece is attached to a conventional anesthetic cartridge with plastic microtubing. However, 0.3-0.4 ml of anesthetic solution are lost within the single-use tube. Another inconvenience of this device is the economic cost of the consumables.

On the other hand, Dentapen® is a wireless device in which the control panel is located in the same main unit (Fig. 3). This allows the clinician to change the injection flow speed in a similar way as with conventional syringes, ensuring maximum autonomy and comfort for the operator. An important advantage of the Dentapen® device is that it does not need specific consumables, since standard dental needles can be used. The Dentapen is supplied with disposable plastic sleeves to prevent contamination from saliva during its use.

The present sample only involved young and mildly anxious women, which may compromise extrapolation of the results. However, Gibson *et al.* (11) and Allen *et al.* (12) have concluded that gender does not seem to be a relevant issue. Another interesting variable is patient anxiety, since it may affect pain perception. Several scales based on questionnaires have been developed to evaluate patient dental anxiety, though the modified dental anxiety scale (MDAS) is the most widely used instrument (13). In our study, 80% of the volunteers had low levels of anxiety (Table 1), probably due to the fact that they were all dental students. Not all reviewed studies have analyzed this parameter. Aggarwal *et al.* (14) found significantly lower anxiety levels when local anesthesia was administered through a C-CLAD system. This could be attributed to the less frightening look of the device compared with the traditional dental local anesthetic syringe. In contrast, Tahmassebi *et al.* (15) and Campanella *et al*. (3) concluded that anxiety levels are independent of the anesthetic device used. A recently published report has also shown that it is not necessary to provide the patient with a detailed explanation of the C-CLAD system, since doing so will not reduce anxiety (16). Hence, if a new clinical trial is made with a sample of more anxious patients, the results would probably be similar, especially taking into account the similarity of the results regarding pain intensity in both groups.

Since pain perception is a highly subjective and variable experience modulated by many factors, several methodologies have been proposed to quantify it (3). The NRS was selected because it is more practical and easier to understand by most people and does not need a paper or pencil (17). Visual analog scales (VAS) are also a reliable alternative, since they are equally sensitive and are superior to the four-point categorical score scale.

A split-mouth design was employed with the objectives of controlling the main confounding variables, to reduce variability and to increase the statistical power. Also, it allowed the contrasting of both procedures in a single session, enabling more direct comparison by the participants. These are very important advantages and, in fact, this methodology was also selected in similar trials performed by Romero-Galvez *et al.* (10), Feda *et al*. (18) and Singh *et al.* (19). However, the study design also has some limitations. Firstly, there was no control group (traditional syringe) and secondly, the first stimulus could condition patient response to the second injection. This particular issue is unlikely to have affected the results in the present study, since the order of the systems was randomized (1:1 ratio).

In conclusion, in most cases (80%), computer delivery controlled-flow injection systems allow the administration of local anesthetics in the palatal area with only mild pain. Both systems (The STA Wand® and Dentapen®) seem to be equally effective in reducing pain perception when palatal injections are needed.
